# The Effect of the Traditional Living Arrangement, Anpakkori, on Depressive Symptoms in Elderly People Residing on Jeju Island

**DOI:** 10.4306/pi.2009.6.3.131

**Published:** 2009-08-22

**Authors:** Eun-Hui Oh, Moon-Doo Kim, Seong-Chul Hong

**Affiliations:** 1 Department of Psychiatry and Institute of Medical Science, Jeju National University School of Medicine, Jeju, Korea.; 2 Department of Preventive Medicine, Jeju National University School of Medicine, Jeju, Korea.

**Keywords:** Depressive symptoms, Correlates, Living arrangement

## Abstract

**Objective:**

We examined the effect of anpakkori, a traditional living arrangement, on depression among elderly people on Jeju Island in Korea.

**Methods:**

A total of 593 subjects were assessed using a sociodemographic questionnaire developed by the authors, the Korean version of Geriatric Depression Scale (KGDS), the Social Support Scale, and the Activities of Daily Living/Instrumental Activities of Daily Living scales (ADL/IADL). Subjects were classified into three groups: those residing with their adult children, those living individually, and those living in the traditional Jeju anpakkori living arrangement.

**Results:**

The prevalence of depression in this study was 53.1%, and the traditional Jeju living arrangement, anpakkori, was significantly correlated with the presence of depressive symptoms (p=0.005)[odds ratio (OR)=1.88, 95% confidence interval (CI)=1.16-3.06].

**Conclusion:**

Living in the traditional Jeju way may not be as good for establishing family solidarity as is living with adult children. Moreover, elderly individuals prone to depression tended to live in this anpakkori living arrangement. Careful psychological and social support systems that might prevent the development of depressive symptoms should be provided for those who live in anpakkori living arrangements.

## Introduction

Korean society is aging rapidly because of a low birth rate and increasing longevity. The percentage of Koreans over the age of 65 was 7.2% in 2000 and 9.1% in 2005.^1^ Population estimates predict that by 2018, 14.5% of Koreans will be over the age of 65, and that number is likely increase to 20.8% by 2026.^2^ The Korean society is aging more rapidly than that of any other developed country.^3^

Given the rapid growth of the elderly population in Korea, the economic and social burdens are likely to pose major public health problems. For example, the 2008 Korean aged welfare budget is 12.2 times greater than the 1998 budget, and the national medical expenses for the elderly were 22.8% greater in 2008 than in 2006.^4^ Aside from the social burden, elderly people themselves experience burdens such as physical illness, functional disabilities, cognitive decline, loss of authority in the family, loss of social position, and financial problems.^5^

Depression is one of the most common medical problems among elderly people.^6^ It has a high recurrence rate and is associated with mortality, poor prognosis, and a lower response rate to antidepressant treatment than in younger age groups.^7^,^8^ In addition, depression in the elderly is associated with an increased risk of functional disability and increased use of health services.^9^,^10^

The prevalence of major depressive disorder in elderly people is about 1%, but rates as high as 15% have been reported for clinically significant depressive symptoms that do not meet the diagnostic criteria for depressive disorder.^11^ The reported prevalence rate of depression in elderly Koreans ranges from 2% to 60%, depending on the diagnostic criteria and methodology used, but it appears to be higher than that for younger people.^12^ Therefore, it is important to identify the variables that affect depression to better understand and prevent this devastating and prevalent illness.

Risk factors for depression in the elderly are: being female,^10^,^13^-^15^ being of older age,^16^,^17^ the absence of a spouse, 13,18,19 low levels of education,^20^,^21^ low socioeconomic status,^15^,^22^ lack of social support,^21^,^23^,^24^ loneliness,^17^,^25^ and poor health.^13^,^16^,^26^ Of these risk factors, low socioeconomic status and loneliness are most commonly associated with depression in elderly individuals.^15^,^17^,^22^,^25^,^27^ From this perspective, it is possible that the traditional Korean extended family could prevent depressive symptoms in the elderly by providing economic support and reducing loneliness. Several studies conducted in Korea have shown that living with adult children increases psychological well-being in elderly people.^28^,^29^ In contrast, most studies conducted in Western countries suggest that living with adult children has a negative psychological effect on the this population^30^,^31^ mainly due to the loss of autonomy and privacy.^32^

Korea has undergone rapid economic development and modernization, and an increasing number of elderly people prefer living independently from their adult children.^33^ Similar to elderly people in Western societies, they report low levels of psychological well-being when living with their children. In those cases, living in the vicinity of, but not in the same house with adult children may be the ideal living arrangement. In this situation, elderly people would receive adequate emotional support from their family while still retaining their autonomy and independence, thus reducing the likelihood of developing depressive symptoms.

Jeju Island in Korea has a traditional living arrangement called anpakkori, in which elderly parents live on the same plot of land as a married son, but in a separate house. In this way, the two families live in the same area, but they have individual households.^34^ According to Lee,^34^ although no definitive and well-established hypothesis about the origin of this living arrangement is known, some agreement has been reached about this phenomenon. First, the culture of Jeju Island involves older parents who are trying to work for as long as their health permits, and who feel disgraced if they rely on their children. Second, under conditions characterized by scarce resources and a poor climate for agriculture and labor, this anpakkori living arrangement in Jeju Island is thought to have been organized to maximize labor power via the efficient organization of family members. The main method for performing agriculture and earning a livelihood in Jeju Island did not involve irrigation, which is labor-intensive, but rather involved dry-field farming and diver fishery, which requires labor on an individual basis. Moreover, women have traditionally formed the majority of the labor force for such work in Jeju Island. Under these circumstances, the anpakkori living arrangement, which is characterized by greater equality between the sexes than is the patriarchal system in a male-dominated society, would make this system advantageous.

To date, no one has studied the effects of this anpakkori living arrangement on psychological well-being and depression in the elderly. In the present study, we examined the effect of anpakkori on depressive symptoms in elderly people living on Jeju Island.

## Methods

### Study population

Our community-based survey was conducted among elderly people aged 65 and older residing on Jeju Island. Non-aboriginal subjects were excluded from the survey because of their cultural differences from the aboriginal subjects. Residents with no children were also excluded. If both of a husband and a wife were available, only one of them was selected for the study. It was not possible to select a random sample because no accurate list of elderly people living with their children in the traditional living arrangement was available. The study sample consisted of three groups: those who lived in the Jeju traditional anpakkori living arrangement, those who resided with their adult children, and those who lived separately from their adult children. After excluding nine participants who did not complete the questionnaires, a total of 593 (193 living in anpakkori, 166 residing with their adult children, and 234 living separately from their adult children) eligible participants were included in the analyses. Written informed consent was obtained from all participants.

### Procedures

The fieldwork was conducted from September 2006 to March 2007. All subjects were interviewed individually by pre-trained interviewers using a standardized questionnaire. Completion of the questionnaire took about 20 min.

### Measurements

#### Sociodemographic Data

Sociodemographic data on age, gender, residential area, marital status, education, self-assessed living standard, employment, and perceived health status were collected on all participants using questionnaires developed by the authors.

#### Assessment of Depressive Symptomatology

The Korean form of the Geriatric Depression Scale (KGDS)^35^ was administered to evaluate depressive symptoms in the subjects. The KGDS was developed from the Geriatric Depression Scale (GDS).^36^ The KGDS consists of 30 "Yes" or "No" questions, scored as 1 or 0, respectively, and has a maximum score of 30. The validity and reliability of the scale were demonstrated previously. 35,37 Jung et al.^35^ suggested that KGDS scores between 14 and 18 be interpreted as borderline or mild depression, scores between 19 and 21 as moderate depression, and scores of 22 or more as severe depression. We defined scores of 18 as indicating the presence of depressive symptoms. Cronbach's α for this study was 0.9.

#### Activities of Daily Living

All respondents were assessed for functional limitations using the Activities of Daily Living (ADL)^38^ and Instrumental Activities of Daily Living (IADL) scales.^39^ The ADL scale consists of five items: eating, getting in and out of the bed, toileting, dressing, and bathing. The IADL scale has six items: climbing a flight of stairs, taking buses, washing or cleaning, shopping, taking medications, and managing finances. The ADL and IADL scales assess the level of dependence for basic activities in daily life. The original items were scored 1-3, but we used a five-point Likert-type scale modified by Yang,^40^ with 1 indicating complete independence, 3 indicating partial need of assistance, and 5 indicated total dependence. The maximum ADL score was 25, and the maximum IADL score was 30. High scores indicated poor functioning. Cronbach's α for this study was 0.90.

#### Social Support

The perceived level of social support was measured by the social support and social conflict items developed by Abbey et al.^41^ Eleven questions measure the affect, affirmation, and aid components of social support. The original items were rated on a 4-point Likert-type scale, but Yoo^42^ revised them to create a five-point Likert-type scale with options that ranged from "very little" to "very much". The total score for this scale was 55, and a high score indicated a high level of social support. Cronbach's α for this study was 0.81.

#### Data Analysis

Differences in proportions were tested using the chisquare test and continuous data were analyzed using an analysis of variance (ANOVA). Bonferroni's correction was used for post-hoc analyses. The correlation between living arrangements and depression was analyzed using a multivariate logistic regression with nine variables (living arrangements, age, sex, residential area, marital status, self-assessed living standard, social support, perceived health status, and ADL score) as independent variables; the presence of depressive symptoms was the dependent variable.

## Results

### Sociodemographic characteristics of the subjects

During the one-year study period, 593 subjects aged 65 years and older complete the questionnaire. The study population consisted of 379 (65.6%) women; 298 (51.9%) subjects lived in Jeju City and 282 (47.9%) subjects lived with a spouse. Of the 593 subjects, 309 (54.4%) were 75 years of age or older, and the average age was 75.33 (SD=7.55). The percent of respondents living with their adult children in anpakkori was 32.5%, whereas 28.0% resided with their adult children, and 39.5% lived separately from their adult children. Most respondents had a low level of education, including 46.7% with no education and 40.4% with fewer than six complete years of schooling. The economic status was reported as "fair" by 380 (68.8%) of the respondents. The percentage of respondents with occupations was 25.8%, and 29.8% reported believing they were healthy (Table 1).

### Comparison of sociodemographic characteristics according to type of living arrangement

Sex (χ^2^=8.33, p=0.016), residential area (χ^2^=31.05, p=0.000), marital status (χ^2^=25.58, p=0.000), education level (χ^2^=22.89, p=0.000), and employment status (χ^2^=14.4, p=0.000) were significantly different among the living arrangement groups. The highest number of respondents living in Jeju City resided with their adult children (62.8%), the fewest lived separately (35.2%), and an intermediate number of subjects resided in the traditional Jeju living arrangement (57.5%). Most subjects living with a spouse resided separately from their adult children (59.3%), with only 33.7% living with their adult children, and 46.4% of the subjects lived in the traditional arrangement. The proportion of subjects with more than six complete years of schooling was the lowest in the traditional living arrangement group (5.2%), highest among those living separately from their adult children (20.2%), and was intermediate (11.4%) among those residing with their adult children. Of the subjects who had an occupation, 15.7% resided with their adult children, 26.4% resided separately from their adult children, and 33.5% lived in the traditional arrangement (Table 2).

### Comparison of age, Korean form of the Geriatric Depression Scale score, social support score, and Activities of Daily Living score among the living arrangement groups

The mean age of the respondents who resided with their adult children was 77.6 years, the mean age of subjects residing separately was 74.6, and the mean age for the anpakkori group was 76.6 years. A significant difference in mean age was found among the three groups (F=9.132, p=0.000). The mean KGDS score was 17.4, 16.8, and 19.2 for respondents living with their adult children, living separately, and in anpakkori, respectively (F=6.635, p=0.001). The mean ADL score was 51.0, 53.5, and 52.0 for respondents living with their adult children, living separately, and living in anpakkori, respectively (F=8.458, p=0.000). No significant difference was found among living arrangement groups on the social support score (Table 3).

### Multivariate logistic regression analysis of depressive symptoms

After adjusting for age, gender, residential area, marital status, self-assessed living standard, social support, perceived health status, and ADL, we found that the traditional living arrangement was significantly correlated with depressive symptoms [odds ratio (OR)=1.88, 95% confidence interval (CI)=1.16-3.06; p=0.011]. We also found statistically significant differences in sex (OR=2.08, 95% CI=1.25-3.47; p=0.005), marital status (OR=1.70, 95% CI=1.04-2.8; p=0.036), self-assessed living standard (OR=3.11, 95% CI=2.04-4.75; p<0.001), and perceived health status (OR=2.99, 95% CI=1.88-4.74; p<0.001) associated with the traditional living arrangement. These data are presented in Table 4.

## Discussion

This study examined the effect of living arrangements on depressive symptoms in elderly people living on Jeju Island. The prevalence of depressive symptoms in the elderly subjects in this study was 53.1%.

Subjects were classified into three groups: those residing with their adult children, those living separately, and those living in the Jeju traditional anpakkori living arrangement. The differences among those groups are shown in Table 2. The group residing with their adult children is characterized as having a high proportion of subjects who are over the age of 75, female, and urban dwellers, who have no spouse, and who are unemployed. The group living in the anpakkori arrangement is characterized by residence in a rural setting, a low educational level, high self-assessed living standard, employed status, and poor perceived health status. The group living apart from their adult children had the highest proportion of subjects under the age of 74; this group was characterized by a higher proportion of males and by participants living with spouses and having higher education, low self-assessed living standard, and poor perceived health status. In summary, the characteristics of the traditional lifestyle group lie in between those of the other two groups. These findings are in agreement with previous reports^43^ indicating that elderly people of an advanced age with poor health and no spouse prefer to live with their children. The traditional living arrangement is a compromise between living with adult children and living separately, and it may be that elderly people whose characteristics lie between those of the subjects that live with their children and those who live separately choose the traditional lifestyle.

The respondents who live in the traditional arrangement reside primarily in the rural area of Jeju Island. This area is less industrialized and urbanized than are other parts of the island, and these subjects may have had fewer educational opportunities than other respondents, which could account for their low economic status. The main industry in this area is agriculture, and these subjects could still have an occupation despite their age. Poor subjective health status can be explained by hard physical labor and the lack of medical facilities.

Several factors such as gender, living arrangements, marital status, self-assessed living standard, and perceived health status were found to correlate with depressive symptoms in this study. Women reported higher levels of depression than did men (OR=2.085, 95% CI=1.247-3.486), and this finding is consistent with those of previous studies.^10^,^13^-^15^ This gender difference may be explained by the fact that women live longer^44^ and tend to have more health problems, more often have no spouse, and have fewer economic resources.^45^

This study found that people who did not have a spouse were more likely to develop depressive symptoms (OR=1.703, 95% CI=1.036-2.798), a finding that is consistent with previous reports,^13^,^18^,^19^ suggesting that loss of a spouse is related to loneliness, decreased help with housework, and a lower economic status, factors that increase the risk for the development of depression.^46^

Subjects who perceived their economical status as low were 3.109 times more likely to have depressive symptoms than were those who felt they had a high socioeconomic status (95% CI=2.037-4.746). This is consistent with previous findings.^15^,^22^ Consistent empirical findings have indicated that older adults who are better off financially experience fewer strains and anxieties and enjoy higher levels of psychological well-being, social integration, life satisfaction, and autonomy.^46^,^47^ Spreitzer and Snyder^48^ reported that the measure of perceived financial adequacy was a more significant predictor of well-being in their elderly subjects than were objective economic factors. Participants who believed that they were in poor health had more depressive symptoms than did those who believed they were healthy (OR=2.990, 95% CI=1.884-4.745). This result is consistent with previous studies that found that health status was an important factor in psychological well-being.^13^,^16^,^26^

In this study, no significant difference in depressive symptoms was found related to age. The relationship between age and subjective well-being varies across studies. The correlation between increased age and the prevalence of depressive symptoms may be the result of the loss of a spouse, lowered health status, or decreased social interaction.^49^ In contrast, some research has demonstrated a positive effect of increased age on psychological wellbeing in elderly people. This can be interpreted as a cohort effect or as a cultural norm in which very elderly people view each day as a bonus.^50^,^51^ Area of residence had no significant effect on depressive symptoms in the elderly. This may be because little difference exists in the living environment or urbanization throughout Jeju Island.

The level of social support did not appear to affect depressive symptoms in this study. No difference in depressive symptoms was found between subjects who reported high levels of social support and those who reported little social support. This finding does not agree with those of most of previous studies.^21^,^23^,^24^ Social support from family and friends helps reduce loneliness,^52^ buffer life stress,^53^ reduce mortality,^54^ lower hopelessness,^55^ and foster better health status.^56^ However, social support does not always have a positive effect and has been associated with negative effects.^57^ Living with adult children may create intergenerational friction and conflict because of differences in lifestyle, attitudes toward the socialization of grandchildren, and inequality in the household division of labor.^58^ A study^59^ conducted on Jeju Island showed that subjective satisfaction, rather than the amount of social support, had a positive effect on quality of life among elderly people. The negative perception group, in which the level of social support was higher than was subjective satisfaction, showed the worst health status and lowest quality of life.

Studies of the relationship between living arrangements and depression in elderly people have reported inconsistent findings. Several studies conducted in Korea revealed that living with adult children had a positive effect on depression in elderly people,^28^,^29^ whereas most studies conducted in Western countries showed that coresidence with adult children had a negative effect on psychological well-being.^30^,^31^ Other studies suggest no relationship between living arrangements and depression.^43^,^60^ These inconsistent findings may be explained by the fact that most of the studies did not control for major factors such as physical, marital, or economical status. Therefore, our study examined the independent effect of living arrangements by conducting a multivariate logistic regression with independent variables of age, gender, residential area, marital status, self-assessed living standard, social support, perceived health status, and ADL score as well as living arrangements.

After adjusting for the independent variables, subjects who lived in the traditional anpakkori arrangement were more likely to have depressive symptoms than were those who resided with their children.

As mentioned in the Introduction, the most likely explanation for this finding might rest on the characteristics of those who lived in the traditional anpakkori arrangement, in that this group was less educated, had worked primarily in agriculture, was not fully independent of their children's financial assistance, and had children who were not able to provide total care for them. In other words, elderly people who were prone to depression tended to live in anpakkori arrangements.

One other feasible explanation for this finding is that relative alienation from their family members' everyday lives might evoke a feeling of loneliness and promote depressive symptoms in elderly people. Loneliness is a specific risk factor for depressive symptoms, and loneliness is positively correlated with depressive symptoms.^61^ Studies^62^ have shown that people living alone often experience more loneliness than do those living with a partner. Moreover, Yang and Hong^63^ reported that living arrangement had a significant effect on loneliness in elderly people, and this effect was not influenced by adding sociodemographic, social activity, or sociopsychological variables.

Living in the traditional Jeju arrangement may be a double-edged sword, however: the elderly parents are not far away enough to feel neglected and are not close enough to lose their autonomy, yet feelings of alienation when they live in the vicinity of their children might increase feelings of loneliness more than if they lived at a distance.

Previous Korean studies have shown that elderly parents who live separately from their adult children but receive financial assistance from them have the highest level of conflict.^64^,^65^ This can be explained, in part, by the fact that as elderly parents become increasingly dependent and support from their adult children increases, the feeling of intimacy and affection of adult children toward their parents decreases.^66^ Choi and Kim^67^ reported that the physical distance between elderly parents and adult children did not affect the perceived conflict with the elderly parents. In contrast, living with adult children lessened the conflict. The most important factor affecting the conflict between elderly parents and adult children was intergenerational solidarity. Though many subjects who lived in the traditional anpakkori arrangement in this study rated their living standard as "fair", 82.8% had an income of less than one million won (about USD $800), and it is likely that these elderly people were financially dependent on their children. If anpakkori is considered living separately as opposed to living with their children, it is possible that these subjects have a high level of conflict with their children caused by financial dependence. However, as neither financial assistance nor level of conflict with adult children was measured by our questionnaire, we cannot determine whether a high level of conflict between parents and adult children existed in this group.

Living with adult children may reduce the risk of developing depression in the elderly, or it may have a positive effect on depression by increasing solidarity between family members, as living with adult children increases perceived solidarity among elderly people.^68^ According to Choi and Kim,^67^ intergenerational solidarity was related to living arrangement, self-esteem, gender, self-control, family support, and economic status. Of those factors, living arrangement was the most important determinant of perceived intergenerational solidarity. Increased solidarity may reduce the risk of depression in elderly people by buffering stress^69^ and easing conflicts with their adult children.^70^ Also, familial solidarity was the most important factor for predicting life satisfaction in elderly individuals.^71^ Indeed, research conducted in Korea showed that elderly people who perceived a high level of familial solidarity had fewer depressive symptoms.^68^ The greater the perceived solidarity with family members was, the less the psychological distress was experienced by elderly people.^72^

In summary, living in the Jeju traditional way may not provide as good an environment for establishing family solidarity as does living with adult children. In addition, because those who were prone to depression tended to reside in this anpakkori living arrangement, psychological and social support systems should be established and provided for this population.

This study has several limitations. We cannot prove a cause-and-effect relationship between living arrangement and depressive symptoms, and our findings might only provide descriptive data. Our questionnaire did not include items that measured loneliness, intergenerational solidarity, and satisfaction with social support with reference to living arrangement. Despite these limitations, the present study provides evidence that living arrangement has a powerful influence on depressive symptoms in the elderly people of Jeju Island.

## Figures and Tables

**TABLE 1:**
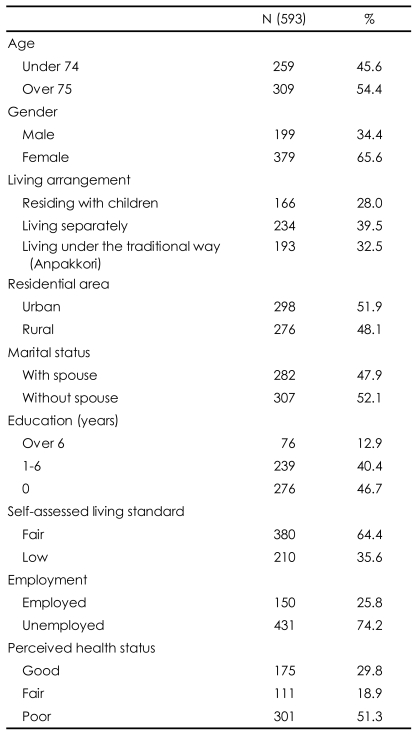
Sociodemographic characteristics of the subjects

**TABLE 2:**
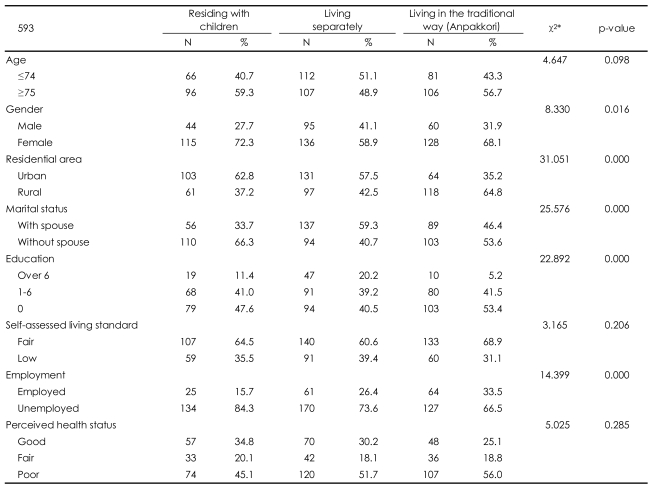
Sociodemographic characteristics according to the living arrangements

^*^χ^2^-test

**TABLE 3:**
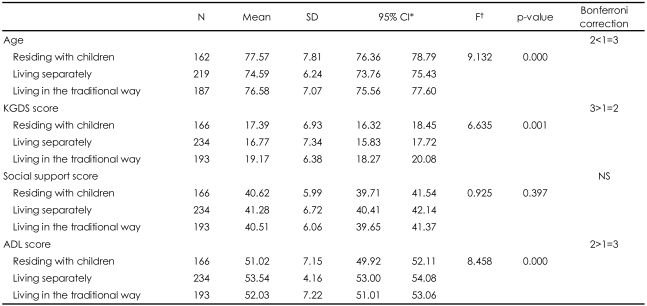
Comparison of age, KGDS score, social support score, and ADL score among the living arrangement groups

^*^Confidence interval for odds ratio, ^†^One-way ANOVA. KGDS: Korean form of the Geriatric Depression Scale, ADL: Activities of Daily Living, NS: not significant

**TABLE 4:**
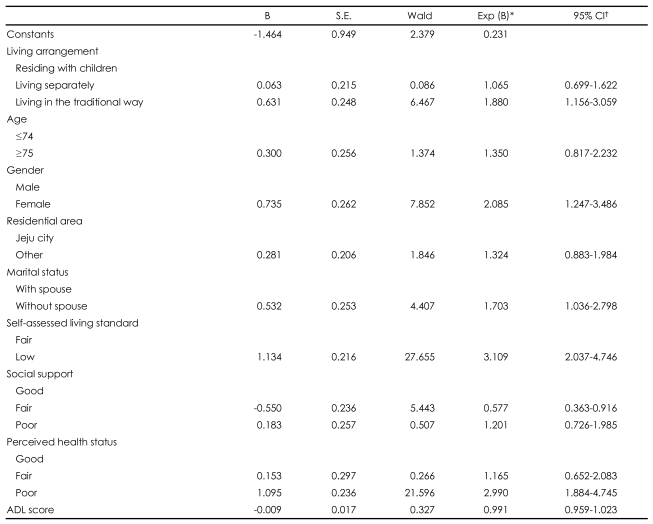
Multivariate logistic regression analysis of depressive symptoms by KGDS score with related variables

^*^Odds ratio, ^†^Confidence interval. KGDS: Korean form of the Geriatric Depression Scale

## References

[1] (2005). Internal migration statistics. Korea Statistics Office.

[2] Population projections for Korea 2005. Korea Statistics Office.

[3] (2005). OECD (Organisation for Economic Co-operation and Development): Economic Survey of Korea.

[4] Population projections for Korea sorted by cities and provinces (2007). Korea National Statistical Office.

[5] Kim JD, Nam CH (1997). Am analysis on determinants of life satisfaction index of the elderly in Korea. Korean Public Health Res.

[6] Minardi HA, Blanchard M (2004). Older people with depression: pilot study. J Advanced Nurs.

[7] Reynolds CF, Frank E, Dew MA, Houck PR, Miller M, Mazumdar S (1999). Treatment of 70(+)-year-olds with recurrent major depression. Excellent short-term but brittle long-term response. Am J Geriatr Psychiatry.

[8] Blazer DG (2003). Depression in later life: review and commentary. J Gerontol A Biol Sci Med Sci.

[9] Beekman AT, Penninx BW, Deeg DJ, de Beurs E, Geerling SW, van Tilburg W (2002). The impact of depression on the well-being, disability and use of services in older adults: a longitudinal perspective. Acta Psychiatr Scand.

[10] Cole MG, Dendukuri N (2003). Risk factors for depression among elderly community subjects: a systematic review and meta-analysis. Am J Psychiatry.

[11] Koenig HG, Blazer DG (1992). Epidemiology of geriatric affective disorders. Clin Geriatr Med.

[12] Kang HS, Kim KJ (2000). The correlation between depression and physical health among the aged. J Korean Public Health Assoc.

[13] Hybels CF, Blazer DG, Pieper CF (2001). Toward a threshold for subthreshold depression: an analysis of correlates of depression by severity of symptoms using data from an elderly community sample. Gerontologist.

[14] Cole MG, Dendukuri N (2003). Risk factors for depression among elderly community subjects: a systematic review and meta-analysis. Am J Psychiatry.

[15] Siegel MJ, Bradley EH, Gallo WT, Kasl SV (2004). The effect of spousal mental and physical health on husbands' and wives' depressive symptoms, among older adults: longitudinal evidence from the health and retirement survey. J Aging Health.

[16] Schoevers RA, Beekman AT, Deeg DJH, Geerlings MI, Jonker C, van Tilburg WJ (2000). Risk factors for depression in later life; results of a prospective community based study (AMSTEL). J Affect Disord.

[17] Adams KB, Sanders S, Auth EA (2004). Loneliness and depression in independent living retirement communities: risk and resilience factors. Aging Ment Health.

[18] Schoevers RA, Beekman AT, Deeg DJH, Jonker C, Van Tilburg W (2003). Comorbidity and risk patterns of depression, generalized anxiety disorder and mixed anxiety-depression in later life: results from the AMSTEL study. Int J Geriatr Psychiatry.

[19] van der Wurff FB, Beekman AT, Dijkshoorn H, Spijker JA, Smits CH, Stek ML (2004). Prevalence and risk-factors for depression in elderly Turkish and Moroccan migrants in the Netherlands. J Affect Disord.

[20] Forsell Y (2000). Predictors of depression, anxiety and psychotic symptoms on a very elderly population: data from a 3-year follow-up study. Soc Psychiatry Psychiatr Epidemiol.

[21] Jang Y, Haley WE, Small BJ, Mortimer JA (2002). The role of mastery and social resources in the associations between disability and depression in later life. Gerontologist.

[22] Kivela SL, Kongas-Saviard P, Kimmo P, Kesti E, Laippala P (1996). Health, health behavior and functional ability predicting depression in old age: a longitudinal study. Int J Geriatr Psychiatry.

[23] Cummings SM, Neff JA, Husaini BA (2003). Functional impairment as a predictor of depressive symptomatology: the role of race, religiosity, and social support. Health Soc Work.

[24] Vanderhorst RK, McLaren S (2005). Social relationships as predictors of depression and suicidal ideation in older adults. Aging Ment Health.

[25] Prince MJ, Harwood RH, Thomas A, Mann AH (1998). A prospective population-based cohort study of the effects of disablement and social milieu on the onset and maintenance of late-life depression. The Gospel Oak Project VII. Psychol Med.

[26] Copeland JR, Chen R, Dewey ME, McCracken CF, Gilmore C, Larkin B (1999). Community-based case-control study of depression in older people. Cases and sub-cases from the MRC-ALPHA Study. Br J Psychiatry.

[27] Kennedy GJ, Kelman HR, Thomas C (1990). The emergence of depressive symptoms in late life: the importance of declining health and increasing disability. J Community Health.

[28] Kwon GS (1973). Familial nuclearization and problems on the aged. Song-Kok Collection of Treatise.

[29] Lim JK (1986). A study on aged populations and social security in Korea. Soc Sec Stud.

[30] Aldous J (1987). New views on the family life of the elderly and the near elderly. J Marriage Fam.

[31] Grams A, Fengler AF (1980). The older parent in the extended family. Presented at the Annual Meeting of the Gerontological Society.

[32] Burch TK, Matthews BJ (1987). Household formation in developed societies. Popul Dev Rev.

[33] Kim CK, Kim S, Hurh WM (1991). Filial piety and intergenerational relationships in Korean immigrant families. Int J Aging Hum Dev.

[34] Lee CG (1999). Population and family in Jeju Island.

[35] Jung IK, Kwak DI, Cho SH, Lee HS (1998). A preliminary study on standardization of Korean form of Geriatric Depression Scale (KGDS). J Korean Neuropsychiatr Assoc.

[36] Yesavege J, Brink T, Rose T, Lum O, Huang V, Adey M (1983). Development and validation of a geriatric depression screening scale: a preliminary report. J Psychiatr Res.

[37] Cho MJ, Bae JN, Suh GH, Hahm BJ, Kim JK, Lee DW (1999). Validation of geriatric depression scale, Korean Version (GDS) in the assessment of DSM-III-R major depression. J Korean Neuropsychiatr Assoc.

[38] Katz S, Ford AB, Moskowitz RW, Jackson BA, Jaffe MW (1963). Studies of illness in the aged. The index of ADL: a standardized measure of biological and psychosocial function. JAMA.

[39] Lawton MP, Brody EM (1969). Assessment of older people: self-maintaining and instrumental activities of daily living. Gerontologist.

[40] Yang YH (1992). Theoretical structural model for the caregiver's role stress and health.

[41] Abbey A, Abramis DJ, Caplan RD (1985). Effects of different sources of social support and social conflict on emotional well-being. Basic Appl Soc Psychol.

[42] Yoo SE (1997). Effects of a predisposition toward perfectionism, social support, and a way of coping with stress on depression of middle-aged women.

[43] Kivett VR, Learner RM (1982). Situational influences on the morale of older rural adults in child-shared housing: a comparative analysis. Gerontologist.

[44] Maddox GL (1979). Sociology of later life. Ann Rev Sociol.

[45] Liang J (1982). Sex differences in life satisfaction among the elderly. J Gerontol.

[46] Larson R, Mannell R, Zuzanek J (1986). Daily well-being of older adults with friends and family. Psychol Aging.

[47] Hughes M, Gove WR (1981). Living alone, social integration, and mental health. Am J Sociol.

[48] Spreitzer E, Snyder EE (1974). Correlates of life satisfaction among the aged. J Gerontol.

[49] Kivett VR (1976). The aged in North Carolina: physical, social and environmental characteristics, and sources of assistance: the Guilford study. Technical bulletin No. 237.

[50] Livingston G, Watkin V, Milne B, Manela MV, Katona C (2000). Who becomes depressed? The Islington community study of older people. J Affect Disord.

[51] Chen CN (2001). Aging and life satisfaction. Soc Indic Res.

[52] Bowling A, Browne P (1991). Social support and emotional well-being among the oldest old living in London. J Gerontol.

[53] Lin N, Woelfel MW, Light SC (1985). The buffering effect of social support subsequent to an important life event. J Health Soc Behav.

[54] Blazer D (1982). Social support and mortality in an elderly community population. Am J Epidemiol.

[55] Park YJ, Lee SJ, Park ES, Chang SO (1999). A prediction model for health promoting behavior of the Korean elderly. J Korean Acad Nurs.

[56] Norbeck JS (1981). Social support: a model for clinical research and application. Adv Nurs Sci.

[57] Thoits PA (1982). Conceptual, methodological and theoretical problems in studying social support as a buffer against life stress. J Health Soc Behav.

[58] Lopata HZ (1973). Social relations of black and white widowed women in a Northern metropolis. Am J Sociol.

[59] Koh BS (2004). A study on the social support types and determinants of perceived quality of life for the elderly in Jeju. J Korean Gerontol Soc.

[60] Fengler AP, Danigelis N, Little VC (1983). Later life satisfaction and household structure: living with others and living alone. Ageing Soc.

[61] Cacioppo JT, Hughes ME, Waite LJ, Hawkley L, Thisted R (2006). Loneliness as a specific risk factor for depressive symptoms in older adults: cross-sectional and longitudinal analyses. Psychol Aging.

[62] Henderson AS, Scott R, Kay DW (1986). The elderly who live alone: their mental health and social relationships. Aust N Z J Psychiatry.

[63] Yang SM, Hong SJ (2003). Living arrangement and psychological loneliness of rural elderly in Korea. J Korean Home Manage Assoc.

[64] Lee YJ (1994). A study on aged parents' conflict with their adult children and depression. J Home Econ Res.

[65] Yoo HJ (2000). Elderly people's perception of the caregiving norms and their relationships with intergenerational solidarity, conflict, and depression. Korean J Gerontol Res.

[66] Cicirelli VG (1983). A comparison of helping behaviors toward the elderly parents of adult children with intact and disrupted marriages. Gerontologist.

[67] Choi JH, Kim TH (1991). Research on aged parent's perception of solidarity and discord with their adult children. J Korean Gerontol Soc.

[68] Kim TH, Kim SJ (1996). A study on the solidarity between the elderly and their three generations, and depression of the elderly. J Korean Gerontol Soc.

[69] Kim TH, Cho HS (1991). A comparison between a mother-and-child family and a husband-wife family on stress-related factors. J Korean Fam Stud Assoc.

[70] Choi JH (1994). Research on adult children's perception of solidarity and discord with their aged parents. J Korean Gerontol Soc.

[71] Cho BE (1990). Intergenerational family solidarity and life satisfaction among Korean aged parents. J Korean Gerontol Soc.

[72] Sussman MB, Robert Binstock, Ethel Shanas (1985). The family life of old people. Handbook of Aging and the Social Sciences.

